# Supplementary material for: Insights into Gene Regulation under Temozolomide-Promoted Cellular Dormancy and Its Connection to Stemness in Human Glioblastoma
Source: Cells. 2023 May 27;12(11):1491. doi: 10.3390/cells12111491 (PMC10252797; doi:10.3390/cells12111491)
Supplement: Supplementary file 1 [file cells-12-01491-s001.zip › Table S1_TaqMan Primers and Probes.pdf]

Supplementary Table 1: TaqMan primer probes (Applied Biosystems, Waltham, MA, USA) used.

Gene	Primer
ACKR4 ≙<br>Chemokine (C-C Motif) Receptor-Like (CCRL)1	Hs00664347_s1
Schlafen (SLFN)13	Hs0111494903_m1
Sloan-Kettering Institute (SKI)	Hs01057032_m1
Cdk5 and Abl Enzyme Substrate (Cables)1	Hs00292828_m1
Dachsous Cadherin-Related (DCHS)1	Hs01044548_m1
Glycerinaldehyde 3-phosphate dehydrogenase (GAPDH)	Hs99999905_m1
Octamer binding transcription factor (OCT)4	Hs00999632_g1
Sex determining region Y-box (Sox)2	Hs00602736_s1
Krueppel-like factor (KLF)4	Hs00358835_m1
