# Supplementary material for: Insights into Gene Regulation under Temozolomide-Promoted Cellular Dormancy and Its Connection to Stemness in Human Glioblastoma
Source: Cells. 2023 May 27;12(11):1491. doi: 10.3390/cells12111491 (PMC10252797; doi:10.3390/cells12111491)
Supplement: Supplementary file 1 [file cells-12-01491-s001.zip › Table S2_Primary antibodies.pdf]

Supplementary Table 2: Primary antibodies used for immunofluorescence staining.

Antibody	Dilution	catalog no.	company
Rabbit anti-CCR11 (≙**CCRL1)**	1:500	#PA5-106552	Invitrogen, Carlsbad, CA, USA)
Rabbit anti-**SLFN13**	1:400	#PA5-54599	Invitrogen, Carlsbad, CA, USA)
Rabbit anti-**SKI**	1:800	#PA5-66852	Invitrogen, Carlsbad, CA, USA)
Mouse anti-**Cables1**	1:50	H00091768-B01P	Abnova, Taipei City, Taiwan
Rabbit anti-CDH10 (≙ **DCHS1)**	1:750	#PA5-101291	Invitrogen, Carlsbad, CA, USA)
Mouse anti-**vWF**	1:1000	sc-53465	Santa Cruz, Dallas, TX, USA
Mouse anti-**CD11b**	1:250	sc-1186	Santa Cruz, Dallas, TX, USA
Mouse anti-**GFAP**	1:500	MAB3402	Merck Millipore, Burlington, MA, USA
Rabbit anti-**OCT4**	1:150	#2750	Cell Signaling, Danvers, MA, USA
Rabbit anti-**Sox2**	1:200	sc-20088	Santa Cruz, Dallas, TX, USA
Mouse anti-**KLF4**	1:250	MA5-15672	Thermo Fisher Scientific, Waltham, MA, USA
