# Supplementary figures and images for: Insights into Gene Regulation under Temozolomide-Promoted Cellular Dormancy and Its Connection to Stemness in Human Glioblastoma
Source: Cells. 2023 May 27;12(11):1491. doi: 10.3390/cells12111491 (PMC10252797; doi:10.3390/cells12111491)

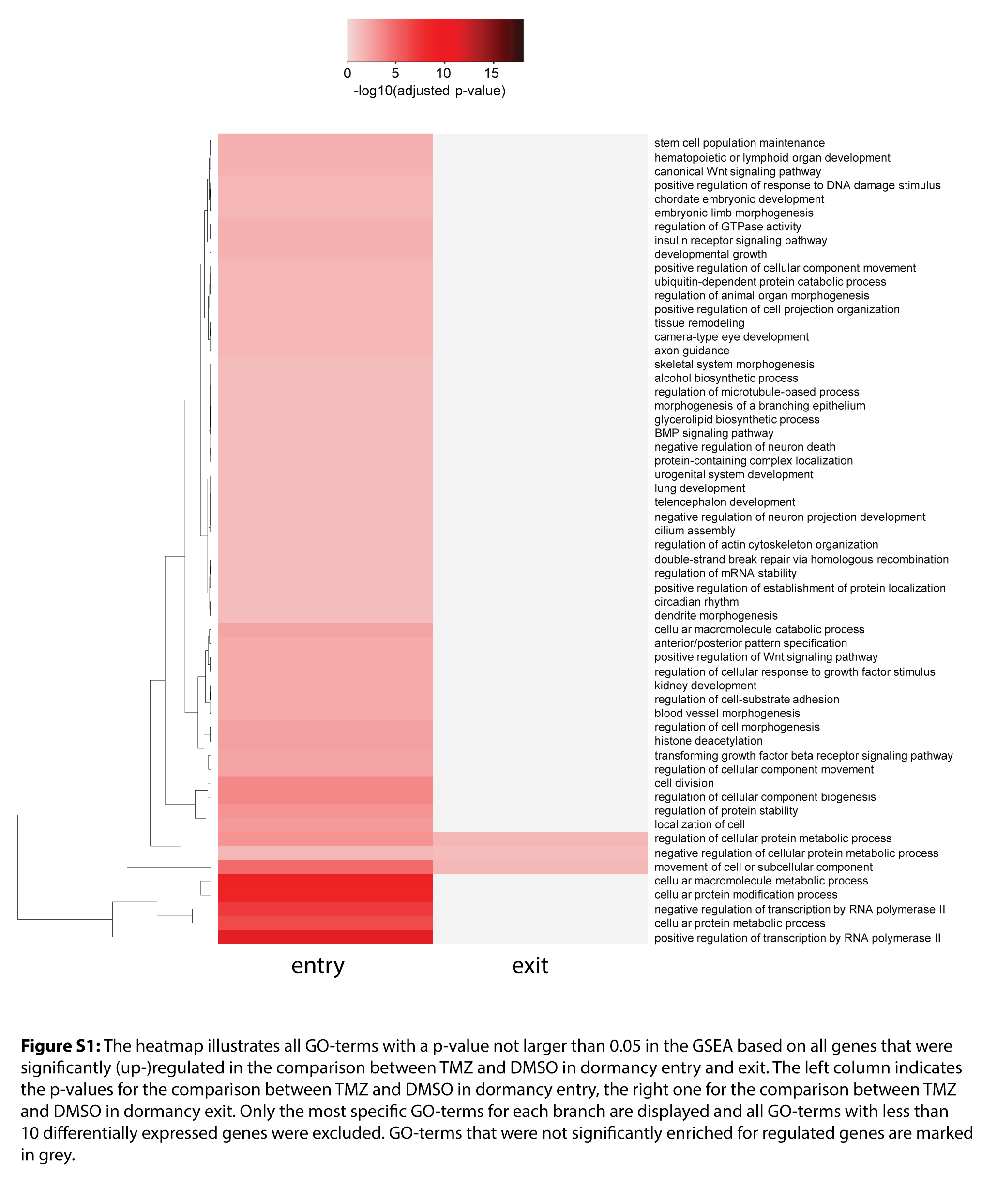

Supplement: Supplementary file 1 [file cells-12-01491-s001.zip › FigureS1.tif]
